# Corticosteroid-Refractory Bullous Pemphigoid Due to Immune Checkpoint Inhibitors: A Telemedicine Challenge

**DOI:** 10.7759/cureus.84911

**Published:** 2025-05-27

**Authors:** Tom Marco, Aaron Bertolo, Rishab Srivastava, Kenneth Barker, Alejandro Recio Boiles

**Affiliations:** 1 Internal Medicine, University of Arizona, College of Medicine – Tucson, Tucson, USA; 2 Internal Medicine, University of Arizona, Tucson, USA; 3 Medicine, University of Arizona, Tucson, USA; 4 Oncology and Hematology, University of Arizona, College of Medicine – Tucson, Tucson, USA

**Keywords:** bullous pemphigoid, clear renal cell carcinoma, drug-induced bullous pemphigoid, immune checkpoint inhibitor adverse effects, immune checkpoint inhibitors, immune-related adverse events, telehealth services

## Abstract

Immune checkpoint inhibitors (ICIs) have become the new standard of care for many malignancies due to their higher efficacy and safer toxicity profile. However, immune-related adverse events (irAEs) can occur unpredictably and in a wide range of organs. Most irAEs present with mild to moderate toxicities. Rarely, severe and life-threatening events occur and can be fatal. Therefore, immediate recognition and management of irAEs are important. Early diagnosis and initiation of treatment with corticosteroids, coupled with diligent follow-up by specialists, are critical steps in optimizing outcomes for patients experiencing irAEs. We present a case report that illustrates the development and progression of severe cutaneous toxicity presenting as a rare bullous pemphigoid rash at the end of two years on nivolumab. It is essential to note that this occurred in an outpatient setting during the COVID-19 pandemic. In coordination with rural providers and telemedicine, the patient’s corticosteroid-refractory ICI toxicity was treated successfully.

## Introduction

The immune system relies on T cells to combat cancer, which can target and eliminate infected or malignant cells. Due to their potency, they can also inadvertently destroy normal cells. To prevent unintended harm to healthy cells, T cells are equipped with specific regulatory mechanisms, often called checkpoints, to temper their activity. By engaging these immunological checkpoints, tumor cells can effectively evade destruction by the immune system [1]. Immune checkpoint inhibitors (ICIs) are immunomodulatory antibodies that enhance the patient’s immune system, allowing the T cells to kill cancer cells. ICIs have emerged as a promising therapeutic approach in recent years, offering new hope in the battle against various types of malignancies such as melanoma, Hodgkin's lymphoma, non-small cell lung cancer, renal cell carcinoma (RCC), urothelial cancer, and liver cancers [2].

Combining tyrosine kinase inhibitors (TKIs) with ICIs has proven more effective in treating advanced RCC, marking a significant advancement in therapy for this condition [3]. While this combination immensely increases survival in cancer patients, it is crucial to acknowledge concerns related to safety and toxicity. Treatment with such drugs can potentially impact any organ system, with the skin, endocrine glands, and gastrointestinal tract being most frequently affected [4]. The pathogenesis of these adverse reactions is currently not fully understood, but multiple proposed theories exist.

Skin manifestations represent the most common immune-related adverse events (irAEs) linked with ICI therapy. The prevalence of all grades of dermatologic toxicity ranges from 17% to 40% for programmed death receptor and ligand 1 (PD-1/PD-L1) inhibitors alone or in combination with anti-cytotoxic T lymphocyte-associated antigen 4 (CTLA-4) or lymphocyte-activation gene 3 (LAG3) antibodies, respectively. In contrast, the incidence of severe dermatologic (grade 4) irAEs varies from 1% to 3% [5,6]. Bullous pemphigoid (BP), a rare autoimmune blistering disorder, represents an uncommon ICI-associated irAE, with reported incidence rates ranging from 0.1% to 1.2% [6]. Early recognition of BP and timely management are essential. With the growing use of telemedicine, particularly in oncology and dermatology, prompt remote evaluation and multidisciplinary coordination can facilitate earlier diagnosis and intervention.

We present a severe case of grade 4 skin toxicity presenting as a rare BP rash at the end of two years on nivolumab (ICI PD-1 inhibitor) and cabozantinib (TKI), complicated by the COVID-19 pandemic in a rural setting. This case illustrates the importance of telehealth and close coordination with other rural specialists to treat severe corticosteroid-refractory ICI toxicity.

## Case presentation

A 65-year-old male with no significant past medical history presented to the emergency room with gross hematuria. A computed tomography of the abdomen and pelvis (CTAP) demonstrated a large right renal mass measuring 11.5 x 9.5 x 9.1 cm in greatest diameter, embracing the inferior margin of the liver without renal vein involvement or capsular extension. A CT of the chest corroborated no definite metastasis to other organs or lymph nodes. Due to having no evidence of distant metastatic disease on imaging, a right radical nephrectomy with adrenalectomy was performed by urology without complications. The pathology report was notable for a clear cell renal carcinoma (10.9 cm), World Health Organization/International Society of Urological Pathology (WHO/ISUP) nuclear grade 2 of 4, staged pT2, and all margins were negative.

As the patient was from a rural area, he had to be referred to a center three hours away for evaluation. The patient was subsequently seen by medical oncology, who calculated the probability of early disease progression to be low at an 8.4% recurrence rate [7]. Given the low-risk recurrence based on the National Comprehensive Cancer Network (NCCN), no adjuvant systemic therapy was recommended [8]. The patient continued to follow up with medical oncology and was under surveillance with CT scans every few months.

At 24 months, a new right upper lobe (RUL) pulmonary nodule measuring 6 mm was found on a non-contrast chest CT. This nodule size was considered non-pathological by the Response Evaluation Criteria in Solid Tumors (RECIST) 1.1 criteria, which assumes a measurable lesion and is potentially pathological if the longest diameter is at least 10 mm when measured by CT or MRI [9]. Furthermore, the lesion was not feasible for biopsy, and the patient continued to be under close surveillance.

At 36 months, the RUL pulmonary nodule measured 10 mm. A new left upper lobe (LUL) nodule measuring 6 mm and a new right lower lobe (RLL) measuring 2 mm were discovered. A follow-up fluorodeoxyglucose PET showed non-avid nodules, and per the tumor board's discussion, it was not feasible to conduct a sampling biopsy.

At 48 months, the RUL pulmonary nodule measured 18 mm, the LUL pulmonary nodule measured 9 mm, and the RLL pulmonary nodule remained 2 mm. The RUL pulmonary nodule measuring 18 mm was now considered pathological size with progression by RECIST 1.1 criteria, and biopsy was recommended. CT-guided biopsy showed pathological confirmation of metastatic carcinoma, consistent with metastasis from the patient’s previously known renal cell carcinoma. The patient was restaged as pT2cN0cM1-IV metastatic renal cell carcinoma with intermediate risk (one point for anemia) and was recommended ICI nivolumab and a TKI cabozantinib following evidence on the CheckMate 9ER Trial [10].

**Figure 1:**
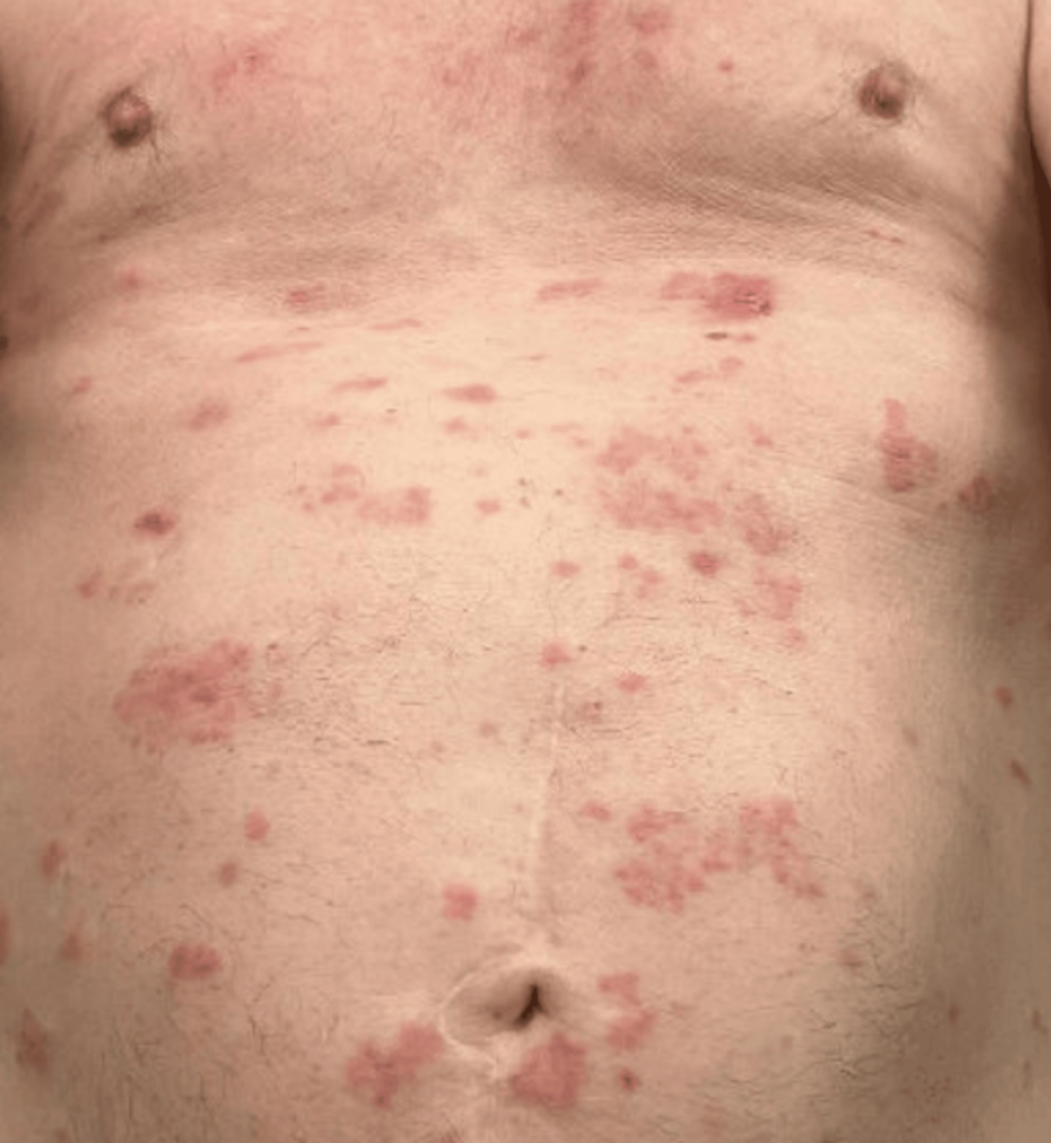
Maculopapular rash, grade I immune-related adverse event.

Shortly after diagnosis, the patient started cycle 1 day one of nivolumab (480 mg every four weeks) and cabozantinib 40 mg daily. After cycle 2, he complained of mild fatigue and rashes that appeared on the anterior chest. Rash was attributed to cabozantinib (TKI) and considered grade I (<10% body surface area (BSA) by irAE grading system). He was treated with topical moisturizer cream and other supportive care recommendations. At this time, imaging showed a partial response of nodules on the RUL and LUL. Following cycle 3, the patient continued to have mild fatigue and rash. The rash was still considered a grade I (<10% BSA) and treated with topical moisturizer cream. In addition, the patient started to have heartburn, gas, and 4 kg weight loss since beginning systemic chemotherapy, which were all considered grade I toxicity, likely related to TKI. Following cycle 5, the patient had a telemedicine call-in-sick visit for his mild rash, which progressed into a diffuse maculopapular rash. The rash was now present on his full back and upper chest (Figure 1). However, it continued to be <10% of BSA and was still considered within grade I. He was prescribed low-potency steroid hydrocortisone cream. The rash was still considered to be more likely related to cabozantinib (TKI) rather than nivolumab (ICI). In addition, cabozantinib likely caused heartburn, gas, and weight loss (4 kg or <10%), all of which are considered grade I toxicity. Imaging showed residual ground glass opacity measuring 4 mm on RUL and LUL. No new pulmonary nodules were identified.

After cycle 9, the patient continued to have the same symptoms and a stable grade 1 rash. Physical findings were as previously mentioned. However, he started to have worsening and significant weight loss (>10% of body weight in the last six months), which was now considered grade 2 toxicity. Due to this, the dose of cabozantinib was reduced to 20 mg daily. Imaging showed that RUL was resolved and LUL had a residual 4 mm lesion.

Subsequent to cycle 10, the patient continued to have similar symptoms as previously mentioned. He also started to have a new mouth sore and a new solitary blistering lesion on the chest (BSA <10%) that did not impact his quality of life. This was still considered grade 1 mucositis and dermatitis, respectively. He was told to continue the hydrocortisone cream on the skin's affected area, continue body moisturizing cream, and start oral rinses with a weak solution of salt and baking soda (one-half teaspoon of salt and one teaspoon of baking soda in a quart of water) for oral mucositis.

Following cycle 13, the patient started to have more blister-like lesions on his chest. One was present on the right upper chest, and one was present on the left lower chest alongside his maculopapular rash that has persisted. In addition, he also had a new open sore on his anus. At this time, the decision was made to stop cabozantinib due to suspected worsening mucositis, dermatitis, and prior gastrointestinal toxicities, affecting his quality of life.

A few days later, due to worsening lesions, the patient presented to his rural hometown primary care physician for a visit. The physician at the time believed these were herpes related skin lesions and started the patient on oral acyclovir. On a follow-up telehealth visit, the patient reported improvement of the rash.

After cycle 14 of nivolumab alone, the patient called and reported having blister-like lesions with open sores on >30% of his BSA despite being off oral TKI for the past two months. This was considered a grade 3 skin irAE likely due to ICI. The decision to hold nivolumab for the 15th cycle was made. He was told to go to the emergency room in his rural hometown. There, he received a one-time dose of Solumedrol 40 mg and was discharged home with prednisone (1 mg/kg/day).

**Figure 2:**
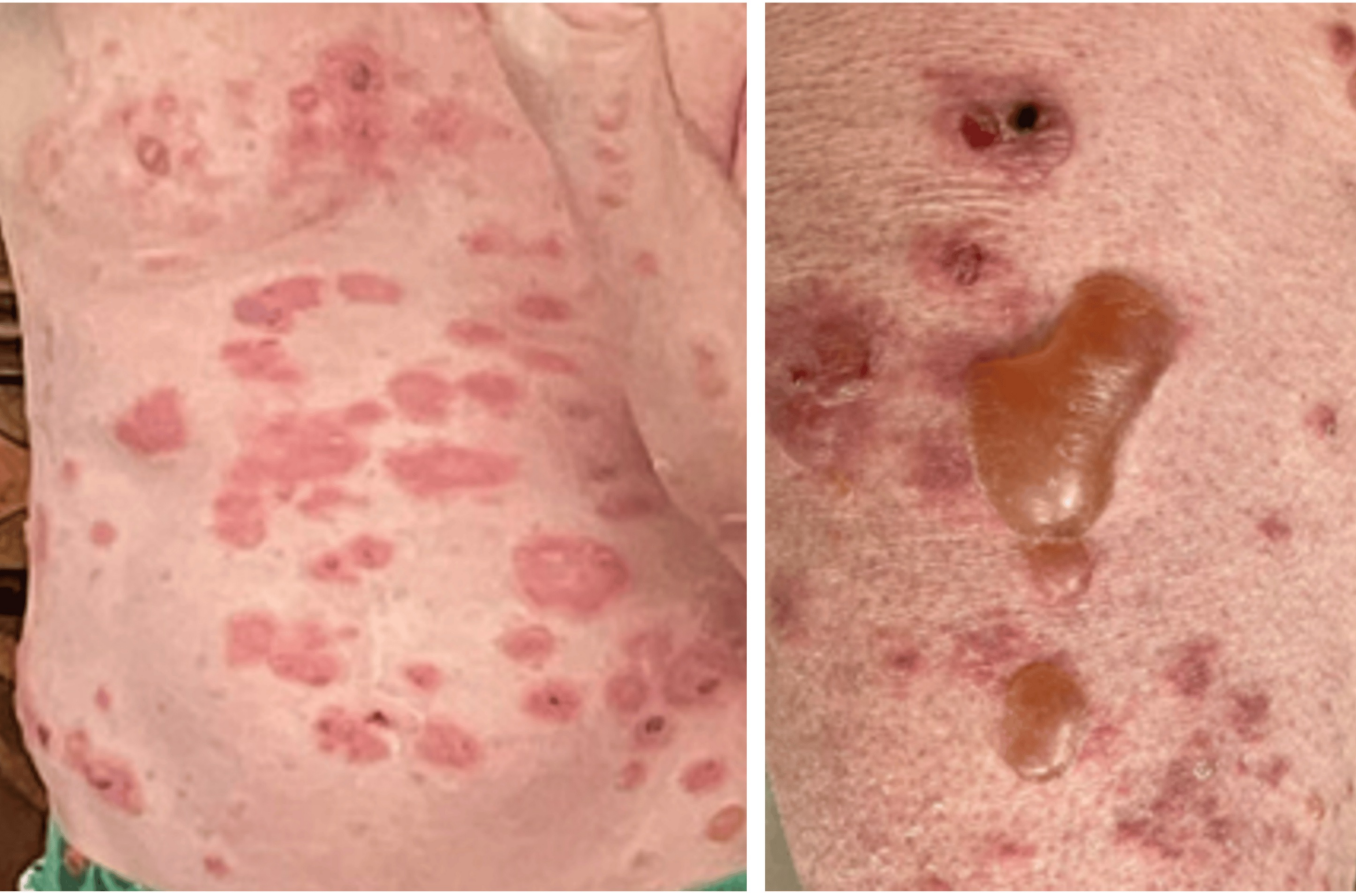
Grade IV immune-related adverse event comprising ≥30% body surface area with bullous lesions.

**Figure 3:**
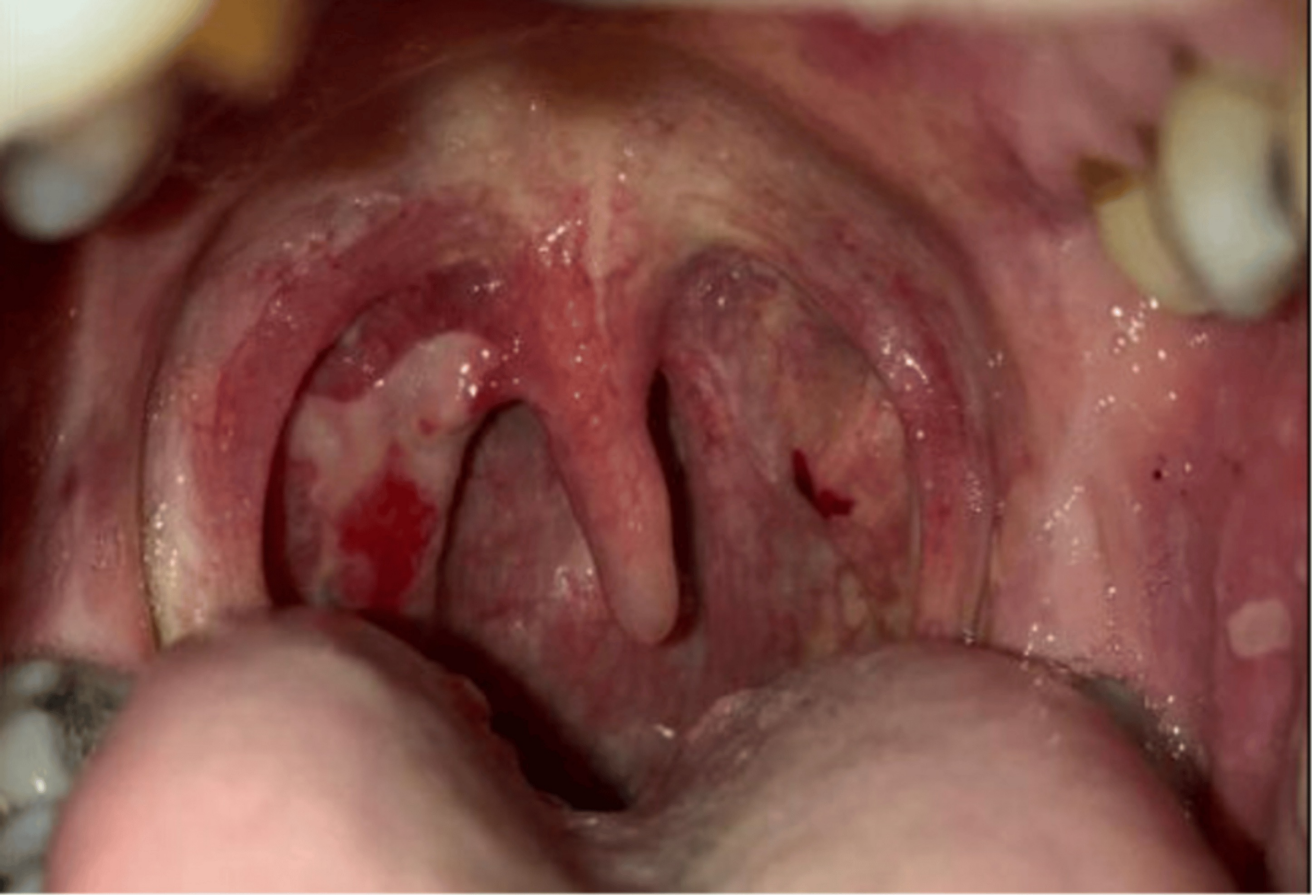
Grade IV immune-related adverse event: oral mucositis.

On day three post 14th cycle, the patient was called and he reported worsening painful rash, and prior blisters progressed to new bullous lesions, affecting more surface areas (Figure 2). In addition, his oral mucositis had also progressed (Figure 3). This was considered a grade 4 severe toxicity (comprising >30% of BSA) with symptoms of bullous/blister lesions, and it was recommended that the patient go to his rural hospital, but he refused hospital transfer at this time. A prescription of prednisone 80 mg (1 mg/kg/day) was sent to his pharmacy.

**Figure 4:**
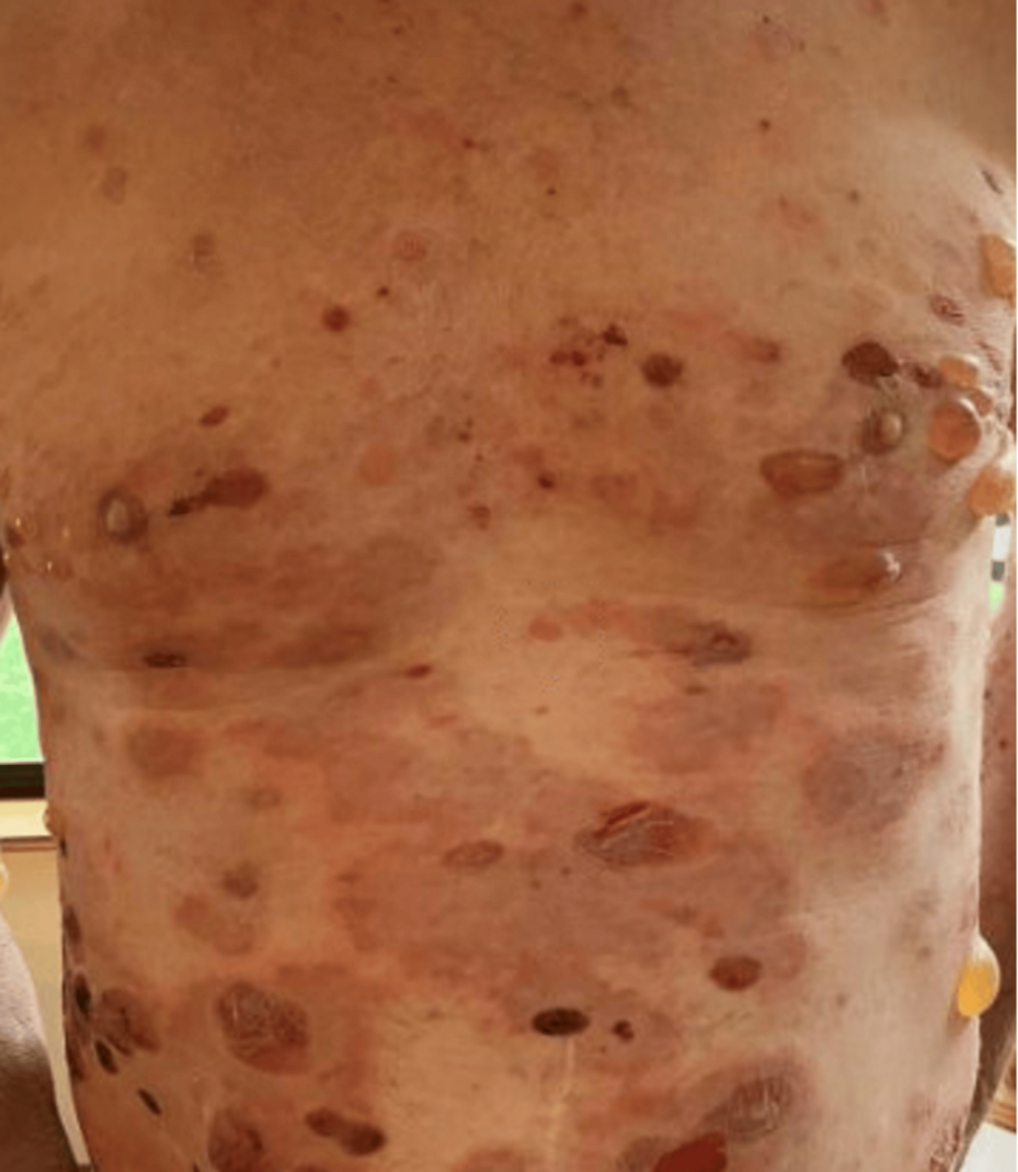
Improved grade IV immune-related adverse event: bullous dermatitis.

Following prednisone, the patient endorsed feeling better. His rash was improved, and he denied new lesions (Figure 4). It was decided to start a slow tapering off the prednisone (decrease by 20 mg every five days). Supportive care for skin (e.g., antimicrobials) and steroid toxicities (e.g., Pneumocystis jirovecii pneumonia and gastritis) was added.

On day seven, the patient presented for follow-up in the office and continued to endorse improvement on the prednisone 40 mg dose. He denied new lesions, and prior known lesions remain extensive (>30% BSA) but were slowly resolving, corroborated by physical exam. He was instructed to extend the prednisone taper and reduce the dose by 5 mg every seven days. He was instructed to report any worsening symptoms immediately.

**Figure 5:**
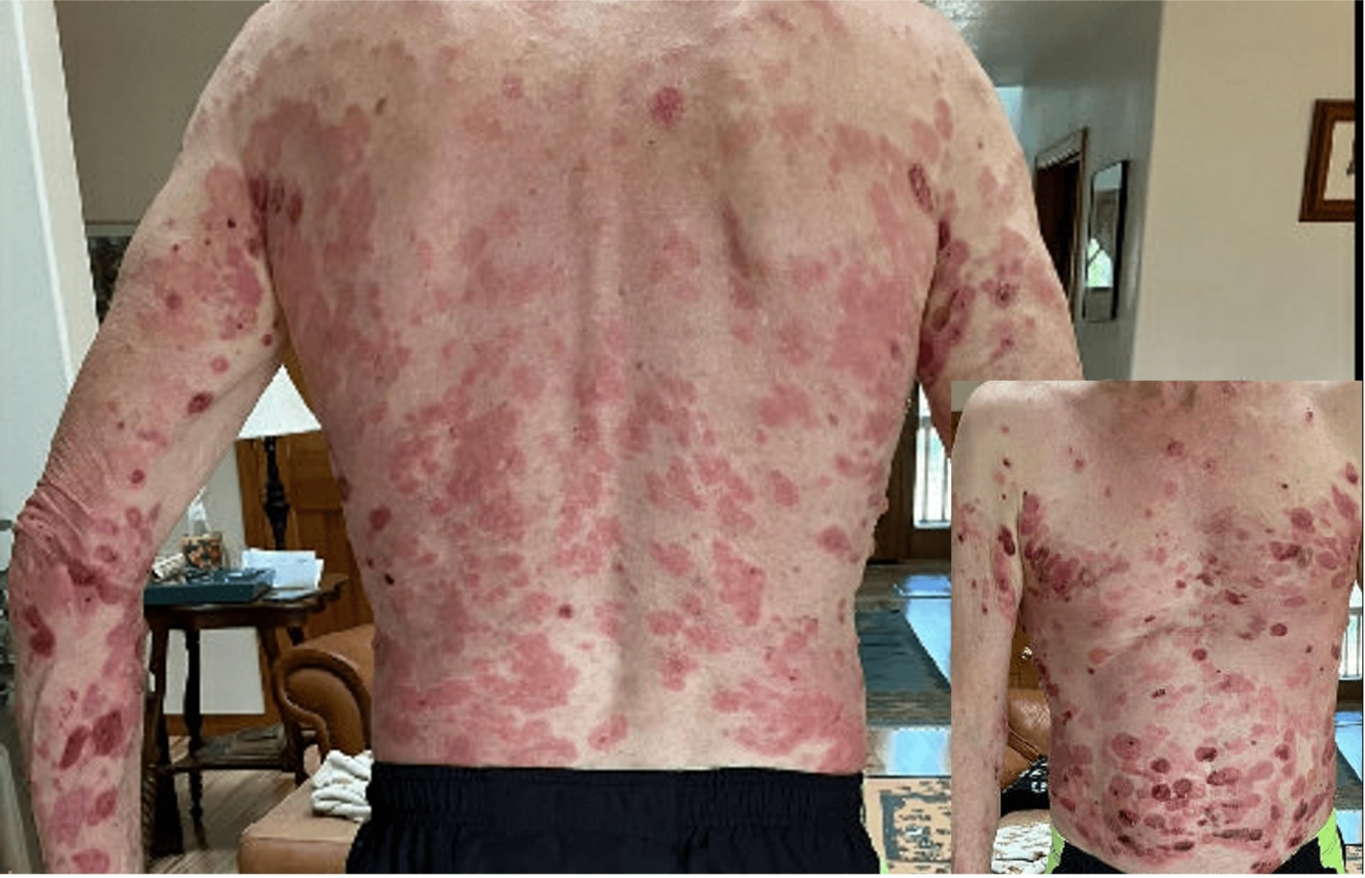
Continued improvement of grade IV immune-related adverse event: bullous dermatitis (front and back).

On day 28, the patient had a telehealth follow-up appointment. He stated feeling overall better as the lesions were drying and reported no new bullae (Figure 5). He endorsed returning to do most of his previous activities, such as biking and hiking, with skin precautions instructions in place. He was instructed to continue prednisone taper and was taking 20 mg daily at the time of this appointment.

**Figure 6:**
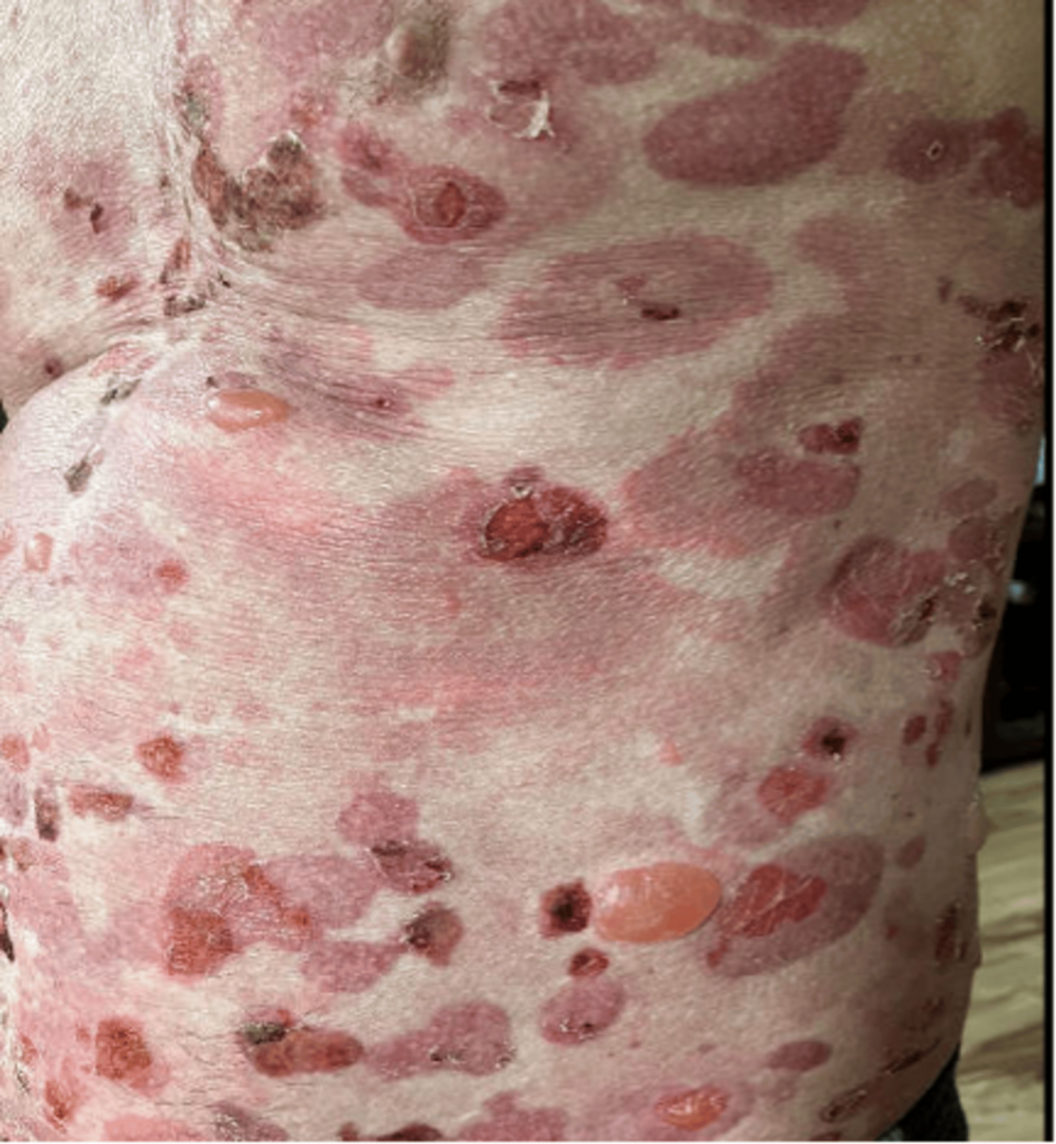
Recurrent grade IV immune-related adverse event: bullous dermatitis (chest/abdomen).

On day 42, he had a flare-up of the bolus lesions (Figure 6) while on 10 mg of prednisone. He was coordinated to be seen by rural dermatology in his hometown and had two punch biopsies done. Pathology reported complete epidermal/dermal separation with loss of the epidermis on the majority of the sections. One tissue section demonstrated linear C3 deposition at the basement membrane zone. There was a small focus of linear IgG noted at the dermoepidermal junction. Based on his pathology results, he was diagnosed with bullous pemphigoid. He was started on doxycycline for 10 days, upgraded on topical steroids to clobetasol with higher potency, and the prednisone dose was increased to 40 mg (0.5 mg/kg/day).

**Figure 7:**
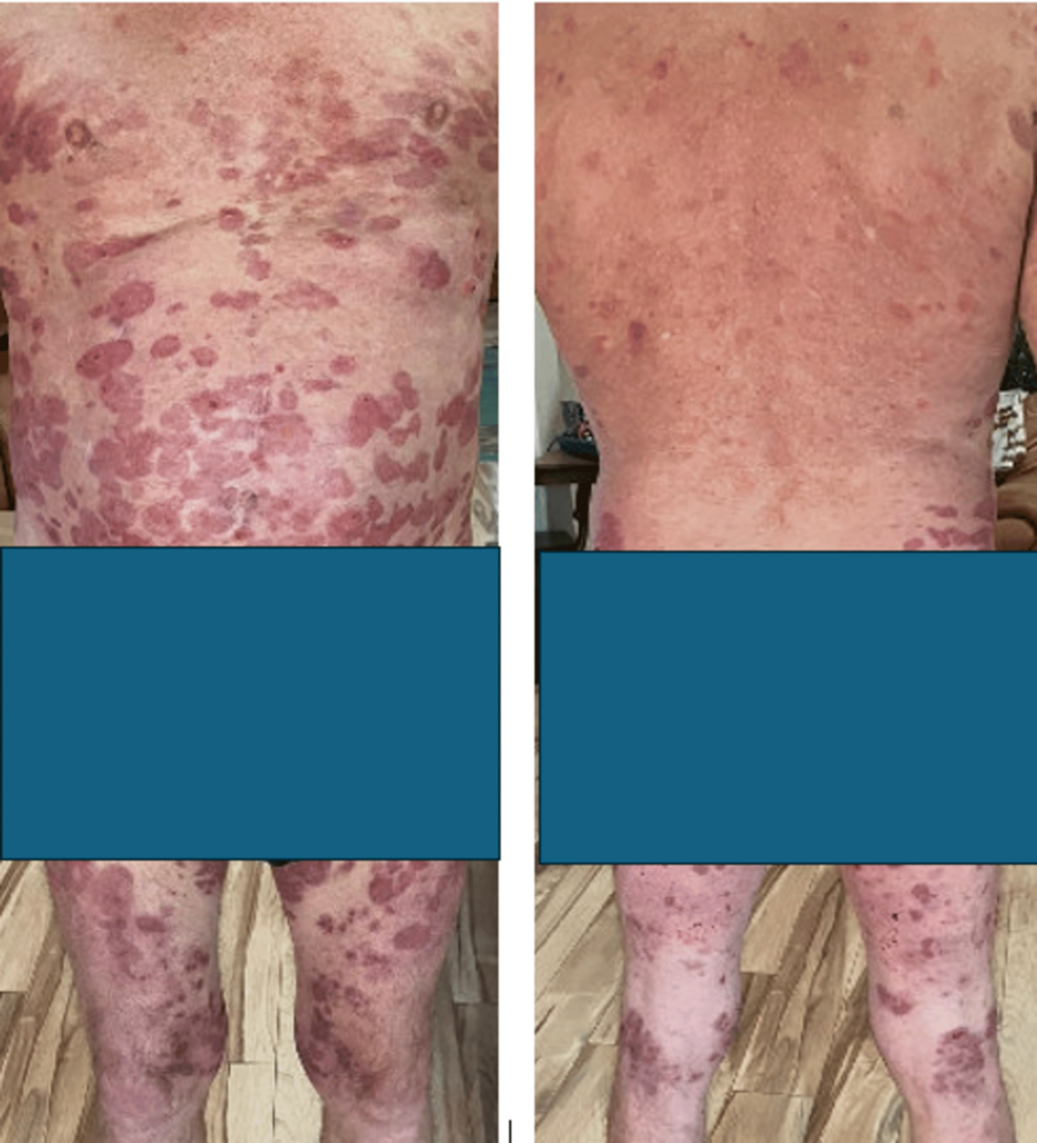
Improved recurrent grade IV immune-related adverse event (bullous dermatitis) following two doses of rituximab (front and back).

On day 63, the patient had a telehealth follow-up appointment with oncology. At the time, the rash improved with an increased dose of prednisone. However, the patient was unable to taper below 30 mg daily due to flares of new lesions. His care was coordinated with dermatology, and a decision was made to start rituximab 375 mg/m2 weekly for two to four doses for recurrent corticosteroid-refractory bullous pemphigoid. After just two doses of rituximab, the patient's lesions showed significant improvement (Figure 7).

On day 91, the patient had a telehealth follow-up appointment. The patient had two doses of rituximab two weeks apart due to the limitations of rural care. He continued to take prednisone and was on 25 mg at the time of the appointment. The patient reported a new blistering lesion above his navel. Oncology discussed with dermatology the benefits of two additional doses of rituximab and further slow, prolonged prednisone tapering.

On day 112, he had an in-person follow-up appointment. The patient completed four doses of rituximab. His rash continued to improve with no new lesions on physical exam. At the time of appointment, he was on 5 mg of prednisone and continued to use topical triamcinolone cream as needed.

**Figure 8:**
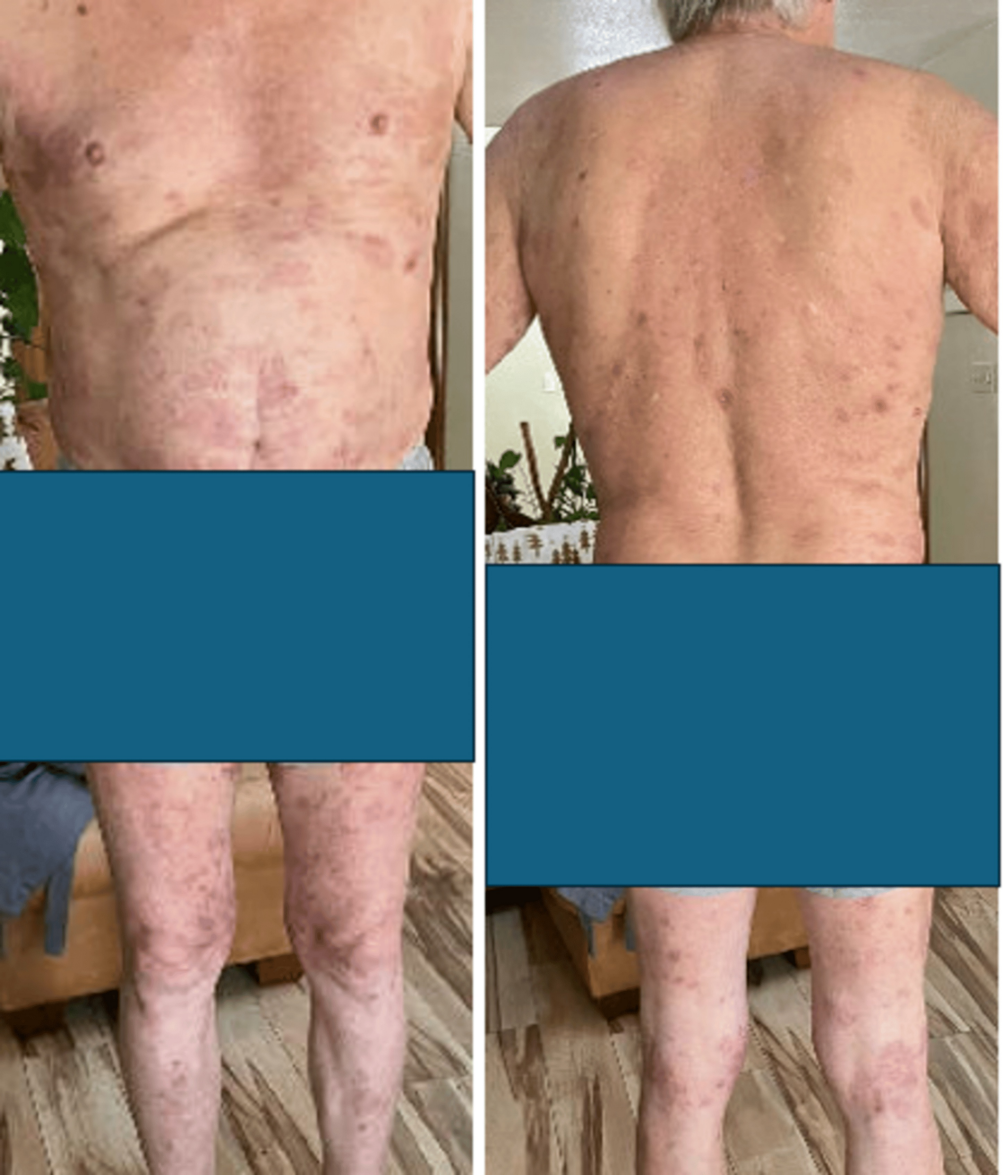
Resolved grade IV immune-related adverse event (bullous dermatitis) following completion of rituximab and prednisone (front and back).

On day 142, the patient had a telehealth follow-up appointment after one month from the last oral steroid dose. The patient stated that he has had no new skin lesions, and the rash was almost completely resolved (Figure 8).

On day 172, the patient returned for an outpatient appointment. He reported no rash, and imaging showed no cancer recurrence. Due to severe and refractory ICI toxicity and balanced with no evidence of disease, the oncology provider decided to start a surveillance protocol (Table 1).

**Table 1:** Quantitative summary of clinical course and toxicity management. BP: bullous pemphigoid; ICI: immune checkpoint inhibitor; RUL: right upper lobe; BW: body weight; BSA: body surface area.

Category	Metric	Value/duration	Notes
Cancer treatment response	Time from ICI start to partial response	~3 months (cycle 1-3)	Imaging showed nodular shrinkage
	Time to confirm metastasis	48 months post-nephrectomy	Biopsy of RUL nodule
	Time on nivolumab + cabozantinib	~10 months (cycle 1-13)	Cabozantinib was stopped due to toxicity
Toxicity onset	Grade 1 toxicity: rash onset	Cycle 2 (~1-2 months)	Maculopapular chest rash
	Grade 2 toxicity: weight loss	Cycle 9 (~6 months)	>10% BW loss
	Grade 4 toxicity: bullous rash onset	Cycle 14 (~9 months)	>30% BSA involvement. Nivolumab discontinued
Steroid therapy	Initial high-dose prednisone	1 mg/kg (80 mg)	Started day 3 post-C14
	Flare on taper	10 mg (day 42 following grade IV toxicity)	Increased prednisone back to 1 mg/kg
Rituximab therapy	Number of doses	4 total	375 mg/m² weekly
Total duration of BP management	From grade 4 onset to resolution	~100 days	Cycle 14 to day 142

## Discussion

The combination of ICI with TKI has become the standard therapeutic approach for the initial management of advanced RCC due to decreased mortality and increased long-term survival rates [10]. Although this combination of medications has positively impacted the treatment of RCC, side effects can be severe.

In this case report, it was initially thought that cabozantinib was causing cutaneous manifestations and gastrointestinal adverse effects. In various studies, it was demonstrated that >70% of patients taking cabozantinib developed one or more cutaneous toxicities encompassing dermatological reactions such as erythema, hand-foot skin reactions with or without blisters, pruritus, seborrheic dermatitis, stomatitis, and xerosis [11,12]. Alternatively, cutaneous manifestations in nivolumab were reported to be around 34% or less and included dermatological toxicities such as maculopapular rash, eczema, lichenoid rashes, vitiligo, pruritis, and rarely, bullous eruptions [13].

Although the dose of cabozantinib was initially reduced and eventually stopped due to multiple suspected toxicities, the patient developed a new, rapidly progressing corticosteroid-refractory rash that continued to worsen to a grade 4 toxicity. Pathology was considered characteristic of an extremely rare BP ICI toxicity. Consequently, the decision was made to discontinue nivolumab. Despite discontinuation of the offending agent, his BP only began to improve after the initiation of rituximab (CD20 inhibitor) in combination with corticosteroids. This suggests that the cutaneous toxicity was attributable to nivolumab, indicating it was an ICI-related adverse effect.

The underlying mechanism of irAEs is not fully understood. Multiple theories exist as to the underlying pathophysiology of these reactions. These include the pro-inflammatory state and uncontrolled activity of T-cells, enhanced immune activation and response, and cytokine release [13]. Skin irAEs are the most common, encompassing 17-40% of toxicities [13] and are assessed and categorized using the cutaneous toxicities from the Common Terminology Criteria for Adverse Events (CTCAE) established by the National Cancer Institute (NCI). The grading system of cutaneous toxicities was published in 2021 by the American Society of Clinical Oncology and spans from 1 to 4, each indicating different levels of severity (Table 2).

**Table 2:** NCI CTCAE v5.0 maculopapular rash and bullous pemphigoid. * Definition: A disorder characterized by the presence of macules (flat) and papules (elevated). Also known as morbilliform rash, it is one of the most common cutaneous adverse events, frequently affecting the upper trunk, spreading centripetally, and associated with pruritus. ** Definition: A disorder characterized by inflammation of the skin, characterized by the presence of bullae, which are filled with fluid. NCI: National Cancer Institute; CTCAE: Common Terminology Criteria for Adverse Events; BSA: body surface area; ADL: activities of daily living.

Adverse event	Grade 1	Grade 2	Grade 3	Grade 4	Grade 5
Rash maculopapular*	Macules/papules covering <10% BSA with or without symptoms (e.g., pruritus, burning, and tightness)	Macules/papules covering 10 to 30% of BSA with or without symptoms (e.g., pruritus, burning, and tightness); limiting instrumental ADL; rash covering >30% BSA with or without mild symptoms	Macules/papules covering >30% of BSA with moderate or severe symptoms; limiting self-care ADL	–	–
Adverse event	Grade 1	Grade 2	Grade 3	Grade 4	Grade 5
Bullous pemphigoid**	Asymptomatic; blisters covering <10% BSA	Blisters covering 10-30% of BSA; painful blisters; limiting instrumental ADL	Blisters covering >30% of BSA; limiting self-care ADL	Blisters covering >30% of BSA; associated with fluid or electrolyte abnormalities; ICU care or burn unit indicated	Death

Most immune-related cutaneous adverse events include maculopapular rashes in grades 1-2 [13]. The recommended treatment includes medium- to high-potency topical corticosteroids applied twice daily until improvement. There is no recommendation to stop therapy at this time. In addition, oral antihistamine therapy can be added in combination to limit pruritus. For those who do not respond to topical steroids and progress to grade ≥3, it is suggested to start systemic corticosteroids (0.5-1 mg/kg/day), tapered over two to four weeks. If the patient remains unresponsive to treatment, alternative treatments such as infliximab and tocilizumab have shown efficacy. Depending on the response to treatment, it may be required to stop ICI therapy [14].

More rarely, those on ICI therapy can develop a BP-like rash. Only 8% of immune-related cutaneous adverse events included BP [15,16]. Grade 1 BP treatment includes high-potency topical corticosteroids applied twice daily until improvement. In grade 2-3 BP, an interruption of ICI therapy is highly recommended. It is suggested to use systemic corticosteroids (0.5-1 mg/kg/day) and supportive infectious treatment with doxycycline (100 mg twice daily). If the patient does not respond, second-line treatments include immunosuppressants (methotrexate or mycophenolate mofetil) or biological agents (such as dupilumab, rituximab, or omalizumab) [15,16]. The patient did not respond to topical and high-dose systemic corticosteroids in this case report. As a result, second-line treatment was required, and rituximab was used.

Severe manifestations (>grade III) are rare, reflecting less than 3% of all occurrences [17]. A four-year retrospective study found that out of 853 patients, only nine (~1%) were identified to develop bullous eruptions [5]. There is limited published data regarding BP induced by ICIs. Most of the literature consists of case reports or small case series from a single medical center. PD-1 inhibitor-associated BP is thought to share a similar pathogenic process with classic BP, involving the production of autoantibodies against hemidesmosomal proteins. However, the specific mechanism related to PD-1 inhibition remains unclear. One article hypothesized that the mechanism is related to PD-1 inhibition, leading to the dysregulation of regulatory B cells, resulting in the nonspecific production of autoantibodies [5]. The diagnosis of BP is confirmed through a combination of clinical features and histopathological and immunohistochemical findings. In the case presented above, the patient began with a grade I rash that progressed to a grade IV toxicity, compromising a painful bullous rash that covered >30% of his body despite the addition of high-potency topical steroid therapy and high-dose oral steroid therapy. He was then referred to dermatology, where a punch biopsy revealed linear C3 deposition at the basement membrane zone and a small focus of linear IgG at the dermo-epidermal junction seen in BP.

As previously mentioned, first-line treatment for grade ≥2 BP toxicities includes using high-potency systemic corticosteroids. When this fails, second-line therapy, including the use of biological agents, is recommended. In a systematic review of 35 publications, 85% of patients who failed steroid treatment were subsequently successfully treated with rituximab. In addition, the recurrence rate was lowest among those treated with rituximab at 29% when compared to the usage of other biological agents [18].

In addition to manifesting a rare and severe skin toxicity associated with ICI therapy, the patient was also at a disadvantage by residing in a rural town located over three hours away from the cancer treatment center during the COVID-19 pandemic, potentially limiting an appropriate treatment in a timely manner. This geographically disadvantaged situation necessitated a coordinated telemedicine approach to patient management involving collaboration between multiple healthcare professionals. Specifically, it required consistent discussions between urban oncology specialists, a small rural primary care practice, a dermatologist, and an emergency department without admission capacity to manage the patient’s care effectively.

This case also illustrates the difficulties faced by cancer patients residing in rural areas. It has been estimated that approximately 19% of the US population lives in rural regions, yet only about 7% of oncologists are based in these areas. This disparity results in a significant proportion of the rural American population lacking access to specialized cancer care, which contributes to increased cancer-related mortality rates [19]. Furthermore, it is estimated that over 36% of individuals living in rural towns must travel long distances to access medical care from physicians managing their conditions [19]. These logistical and access barriers highlight the critical need for telemedicine for those residing in rural towns to improve cancer care accessibility compared to the currently available approaches. As demonstrated in this case, telehealth medicine can be one of multiple solutions. Due to the severity of this patient’s toxicity, weekly follow-ups with a trained oncology specialist were crucial. However, the patient could not travel over six hours per day every week for appointments. Due to the COVID-19 pandemic and the popularization of telehealth medicine, the patient was followed closely by his medical oncologist. Although he was regularly monitored through telehealth, due to his worsening toxicity, he was advised to go to an ER in his rural hometown that did not have the capability or specialist services to treat his condition correctly. The limited exposure and experiences of rural healthcare providers with ICIs necessitate effective communication and education from specialists as these medications become more commonly used. Potential solutions involve establishing partnerships between urban and rural healthcare providers and developing outreach programs that bring specialized services closer to underserved communities.

## Conclusions

The development of cutaneous toxicities from ICI therapy presents a significant challenge, particularly for rural oncology patients. This case highlights a rare, severe skin reaction complicated by the patient’s distance from care. Managing these toxicities is difficult due to their diverse manifestations, which mimic other conditions like herpes zoster and eczema, delaying diagnosis and treatment. Rapid progression can lead to life-threatening complications, including infection and sepsis. Worsening toxicity may necessitate halting therapy, disrupting treatment. This case also underscores how a telehealth approach can ensure continuous care even in geographically isolated regions. The patient was able to receive cancer treatment, undergo detailed toxicity monitoring, and benefit from multidisciplinary input without a significant travel burden. The early detection and management of high-grade toxicities through remote assessments and care coordination ultimately enabled successful treatment outcomes. Given this, structured telehealth surveillance protocols are crucial and can serve rural areas that lack specialty centers.
